# The potential role of non-coding RNAs in acute kidney injury: a focus on natural medicine treatment

**DOI:** 10.3389/fmolb.2025.1648526

**Published:** 2025-08-07

**Authors:** Yuxin Guo, Yanru Zhao, Hanqing Zhang, Yunze Xing, Yaxuan Fang, Dingyuan Zheng, Hanqi Yang, Yanheng Qiao, Bo Yang

**Affiliations:** ^1^ Department of Nephropathy, First Teaching Hospital of Tianjin University of Traditional Chinese Medicine, Tianjin, China; ^2^ Department of Nephropathy, National Clinical Research Center for Chinese Medicine Acupuncture and Moxibustion, Tianjin, China

**Keywords:** acute kidney injury, non-coding RNA, natural medicine, biomarkers, LncRNA-long noncoding RNA

## Abstract

Acute kidney injury (AKI) is a complex clinical disease characterized by sophisticated molecular pathways. Non-coding RNAs (ncRNAs), including lncRNAs, miRNAs, and circRNAs, play a pivotal role. This review focuses on the role of ncRNAs in AKI pathogenesis and their potential as biomarkers for early detection and therapeutic intervention. We carried out an extensive examination of the expression patterns, functional roles, and molecular mechanisms of lncRNAs, miRNAs, and circRNAs in AKI, as well as their relevance to treatments with natural medicines. Our findings underscore the dualistic nature of ncRNAs in AKI, acting as both protective and detrimental factors that influence key biological processes includes inflammation, oxidative stress, and apoptosis. We also point out the possible importance of other ncRNA types, such as snoRNAs and snRNAs, in AKI pathophysiology. This review provides a holistic perspective on the role of ncRNAs in AKI and lays the theoretical groundwork for the creation of innovative therapeutic strategies and biomarkers.

## Graphical Abstract

**Figure:**
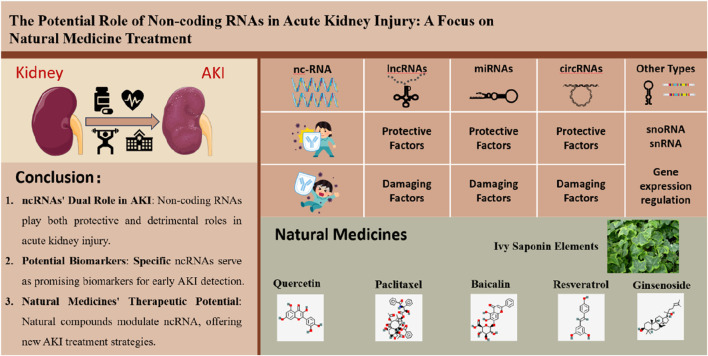
The figure illustrates the potential role of non-coding RNAs (ncRNAs) in acute kidney injury (AKI) and their relevance to natural medicine treatment. On the left side, it shows a kidney and AKI, with key conclusions listed below, including the dual role of ncRNAs in AKI, their potential as biomarkers, and the therapeutic potential of natural medicines. The right side categorizes ncRNAs into lncRNAs, miRNAs, circRNAs, and other types like snoRNAs and snRNAs, highlighting their protective and damaging factors. At the bottom, it displays natural medicines such as Ivy Saponin Elements, Quercetin, Paclitaxel, etc., indicating their therapeutic potential in AKI by modulating ncRNAs.

## 1 Introduction

Acute kidney injury (AKI) occurs when blood creatinine levels rise rapidly in a short time, often with symptoms like oliguria or anuria. It is associated with renal damage, an elevated risk of coronary artery disease and chronic renal disease, affecting around 20% of hospitalized patients (Peerapornratana et al., 2019). Despite this significant incidence, AKI management is often inadequate, and reliable early identification markers are lacking. Thus, it is essential to pinpoint effective biological markers for AKI’s onset and progression for timely intervention. Non-coding RNA (ncRNA), which is a potential therapeutic target and diagnostic biomarker for AKI, is increasingly shown to be important in this process. The goal of this paper is to give a thorough overview of ncRNAs in AKI, focusing on lncRNAs, circRNAs, and miRNAs, and evaluates their potential to serve as trustworthy biomarkers for the condition.

Non-coding RNAs (ncRNAs) have emerged as pivotal regulators in various biological processes, with lncRNAs, circRNAs, and miRNAs being the most extensively studied types. These ncRNAs have been well-documented to play crucial roles in kidney physiology and pathophysiology, making them promising candidates for biomarker discovery and therapeutic development. Accumulating evidence has highlighted their involvement in the complex mechanisms underlying AKI. For instance, lncRNAs have been reported to regulate kidney cell proliferation and apoptosis, circRNAs have been implicated in modulating renal inflammatory responses, and miRNAs have been linked to kidney fibrosis and repair processes (Chang et al., 2022; Ma et al., 2023; Jiang et al., 2020). In light of this growing body of evidence, we focus on these three types of ncRNAs to provide a comprehensive understanding of their roles in AKI and to explore their potential clinical applications.

## 2 Classification and role of noncoding RNAs

Advancements in high-throughput sequencing have increased interest in ncRNAs, which make up over 90% of RNAs in the human genome. They can be classified into structural and regulatory categories. Small ncRNAs are defined as those under 50 nucleotides, including small interfering RNAs (siRNAs), microRNAs (miRNAs), and PIWI-interacting RNAs (piRNAs). Intermediate-length ncRNAs include small nucleolar RNA (snoRNA) and small nuclear RNA (snRNA). Circular RNAs (circRNAs) have a closed-loop structure, whereas long non-coding RNAs (lncRNAs) are longer than 200 nucleotides (Good, 2023). Compared to messenger RNAs (mRNAs), ncRNAs are often more crucial in disease development. Acting as competitive endogenous RNAs (ceRNAs), they influence various physiopathologic processes; for example, circPDK1 enhances glucose metabolism in pancreatic cancer by sponging miR-628-3p (Lin et al., 2022). Moreover, ncRNAs can interact with proteins; lncRNA SNHG17 stabilizes c-Myc proteins, promoting cell proliferation (Liu J. Y. et al., 2021). NcRNAs also influence protein translation, with modifications such as N6-methyladenosine affecting circRNA function in response to cellular stress, which may impact miRNA interactions (Wang X. et al., 2021). Recent studies suggest some lncRNAs and circRNAs may also perform translational functions, marking a new area of research (Wu et al., 2020). Their diverse roles extend to various diseases, including renal disease, cancer, cardiovascular disease, diabetes, and autoimmune disorders.

## 3 ncRNA and AKI

**FIGURE 1:**
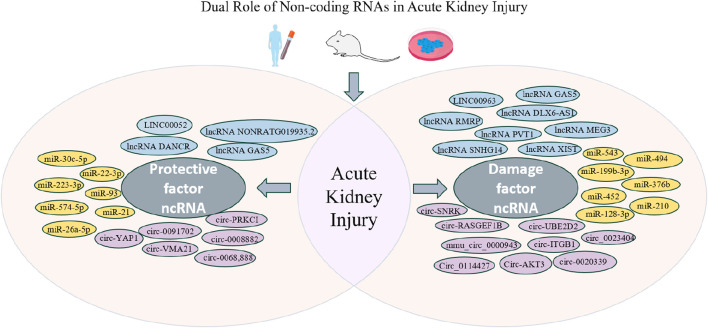
Dual Role of Non-coding RNAs in Acute Kidney Injury. The figure shows the dual role of non-coding RNAs (ncRNAs) in acute kidney injury (AKI). On the left are protective ncRNAs like lncRNA, miRNA, and circRNA that help reduce kidney damage and aid recovery. On the right are damaging ncRNAs that may worsen kidney injury and contribute to AKI’s progression. It lists specific ncRNAs and their roles in AKI. (Created with FigDraw).

Recent studies have emphasized the regulatory functions of non-coding RNAs (ncRNAs) in the transcription and translation of proteins, with a particular focus on their altered expression patterns in sepsis-induced acute kidney injury (SA-AKI). Evidence suggests that abnormal levels of lncRNAs, miRNAs, and circRNAs play a role in the development and course of AKI. This paper summarizes current research on these ncRNA types, describing the molecular mechanisms, functional roles, and expression patterns of each. NcRNAs are anticipated to become novel therapeutic targets or biomarkers for early detection and prognostic assessment of AKI (Figure 1).

### 3.1 lncRNA

Initially, lncRNAs were considered transcriptional noise from RNA polymerase II due to their lack of an open reading frame (Uszczynska-Ratajczak et al., 2018). However, high-throughput sequencing has revealed their significance at various embryonic stages. The subcellular localization of lncRNAs determines their functional roles: nuclear lncRNAs regulate mRNA translation, splicing, localization, and transcription, while cytoplasmic lncRNAs can act as “miRNA sponges” to control mRNA degradation (Gil and Ulitsky, 2020). It's getting clearer how lncRNAs function in the pathophysiology of AKI. As summarized in Table 1, we classified findings from studies on lncRNAs in AKI into protective and detrimental factors based on their effects.

**TABLE 1:** The possible molecular mechanisms and functional roles of lncRNA in AKI.

lncRNA	Sample type	Expression	Functional role	Signaling pathway/target gene	References
DANCR	Serum and cellular	Downregulates	Increases cell viability and prevents inflammatory reaction and apoptosis caused by LPS	miR-214/KLF6	Zhao et al. (2020)
GAS5	Kidney tissues and cells	Downregulates	Inhibits cellular pyroptosis	miR-579-3p/SIRT1/PGC-1alpha/Nrf2	Ling et al. (2021)
GAS5	Kidney tissues and cells	Upregulates	Promotes apoptosis	miR-1/TSP-1	Geng et al. (2020)
9935	Kidney tissues and cells	Downregulates	Inhibits p53-mediated apoptosis	HuR/Tp53	Ding et al. (2021)
LINC00052	Kidney tissues and cells	Downregulates	Promotes cell proliferation and inhibits hypoxia-induced apoptosis	miR-532-3p/Wnt/β-linker protein	Li et al. (2020)
DLX6-AS1	Serum and cells	Upregulation	Releases inflammatory factors and promotes cellular pyroptosis	miR-223-3p/NLRP3	Tan et al. (2020)
RMRP	Serum and cellular	Upregulates	Promotes LPS-induced apoptosis and inflammatory response	NLRP3/miR-206/DDX5	Zhang et al. (2021a)
PVT1	Kidney tissue, serum and cells	Upregulates	Inhibits cell viability and promotes cellular pyroptosis, apoptosis and inflammatory responses	miR-20a-5p/NLRP3 miR-17-5p/NF-κB	Deng et al. (2021), Yuan et al. (2021)
SNHG14	Kidney tissue, serum and cells	Upregulation	Inhibits cell viability and promotes inflammation and oxidative stress	miR-124-3p/MMP2	Xue et al. (2021)
XIST	Kidney tissues and cells	Upregulates	Delays cellular damage	miR-142-5p/PDCD4	Tang et al. (2020)
LINC00963	Kidney tissues and cells	Upregulates	Promotes apoptosis	miR-128-3p/JAK2/STAT1	Xie et al. (2020)
MEG3	Cells	Upregulates	Activates cellular autophagy and induces apoptosis	MEG3/miR-145-5p/RTKN/Wnt/β-catenin/c-MYC	Liu et al. (2021b)

#### 3.1.1 lncRNAs as protective factors

Multiple studies report altered lncRNA expression in AKI contexts. Evidence quality varies across experimental models. Human biomarker studies show that DANCR serum levels are downregulated in AKI patients versus controls. Zhao et al. reported that DANCR “sponges” miR-214 *in vitro* (Zhao et al., 2020). Its clinical utility as a biomarker requires validation of sensitivity and specificity. LINC00052 plasma levels are reduced in AKI patients. Li et al. found that LINC00052’s correlation with hypoxia and Wnt/β-catenin signaling remains unvalidated in human tissues (Li et al., 2020).

Rodent models provide mechanistic insights. GAS5 is downregulated in SA-AKI mouse kidneys and LPS-HK2 cells. Ling et al. observed that this correlates with cell death via the miR-579-3p/SIRT1 axis (Ling et al., 2021). However, Geng et al. discovered that GAS5 is upregulated in I/R-AKI kidneys and promotes apoptosis via miR-21/TSP-1 (Geng et al., 2020). This discrepancy highlights model dependence. NONRATG019935.2 (9935) is downregulated in SA-AKI rat kidneys. Ding et al. demonstrated that overexpression of 9935 prevents kidney tubular epithelial cells from undergoing apoptosis. It interacts with p53 and stabilizes Tp53 mRNA (Ding et al., 2021).

Critical knowledge gaps remain. No established lncRNA “signature” for AKI staging exists due to fragmented studies. The origin of circulating lncRNAs, such as DANCR and LINC00052, is unclear. They may result from passive leakage due to kidney damage or active secretion. Therapeutic modulation, such as LINC00052 overexpression in rodents, lacks *in vivo* delivery strategies and safety data.

#### 3.1.2 lncRNAs as damaging factors

Conversely, certain lncRNAs exhibit elevated expression in AKI and may drive inflammation and renal damage (Table 1). Evidence from human studies provides initial biomarker insights. In SA-AKI patients, serum DLX6-AS1 levels rise and correlate with creatinine—a clinical indicator of kidney dysfunction (Tan et al., 2020). This suggests DLX6-AS1 could serve as a liquid biopsy biomarker. Similarly, RMRP increases in patient serum and LPS-exposed HK-2 cells. When silenced, RMRP reduces inflammation and cell death in mice, hinting at its pathogenic role (Zhang X. et al., 2021).

Animal and cellular models reveal mechanistic actions. PVT1, overexpressed in SA-AKI mouse kidneys and human serum, “recruits” miR-20a-5p to activate NLRP3, fueling pyroptosis and inflammation (Deng et al., 2021; Yuan et al., 2021). In HK-2 cells, SNHG14 acts as a miR-124-3p “sponge” to elevate MMP2, worsening ischemia-reperfusion injury (Xue et al., 2021). XIST, upregulated in I/R-AKI, suppresses miR-142-5p and elevates PDCD4—a cell death promoter (Tang et al., 2020).

Controversies and knowledge gaps persist. LINC00963 targets miR-128-3p to block apoptosis in AKI (Xie et al., 2020), while MEG3 activates Wnt/β-catenin to exacerbate injury (Liu D. et al., 2021). These opposing roles highlight context-dependent functions. Most mechanisms (e.g., PVT1/NF-κB (Yuan et al., 2021)) are cell-derived; their relevance in human tissues remains unverified. Critically, these lncRNAs are detected in blood, but further research is needed to determine whether they originate from damaged kidneys or actively signal disease progression. Future studies should focus on validating these lncRNAs in larger human cohorts and exploring their potential as therapeutic targets.

### 3.2 miRNA

MiRNAs target messenger RNAs (mRNAs) and regulate their expression, playing a crucial role in various pathogenic diseases. Changes in miRNA expression levels reflect disease activity and can serve as biomarkers for diagnosing conditions such as inflammation and cancer (Condrat et al., 2020). Studies have confirmed alterations in serum and urine miRNA levels in acute kidney injury (AKI), suggesting their potential as biotherapeutic agents and their significance in AKI development. Monitoring miRNA expression could aid in the identification and management of AKI (Mahtal et al., 2022).

#### 3.2.1 miRNAs as protective factors

In the context of AKI, blood-derived and tissue-derived miRNAs reach renal tissues, binding to target genes to suppress apoptotic signals and inflammatory mediators. In the rat model of SA-AKI, miR-22-3p is significantly diminished, reducing inflammation and apoptosis by targeting PTEN (Wang X. et al., 2020). Zhang et al. found that SA-AKI patients have downregulated miR-22-3p in serum and urine, with a negative correlation between miR-22-3p levels and renal impairment indicators, suggesting it as a prognostic biomarker for 28-day survival (Zhang H. et al., 2021).

miR-93 protects kidney tubular epithelial cells from LPS-induced apoptosis via the PTEN/AKT/mTOR pathway (Zhan et al., 2021). Antioxidant miRNAs like miR-30c-5p and miR-223-3p inhibit cellular pyroptosis (Li et al., 2021; Gao M. et al., 2022). However, Liu et al. found no association between renal damage biomarkers and miR-574-5p, indicating that miRNAs could aid in AKI diagnosis and management (Liu S. et al., 2021).

In bone marrow-derived dendritic cells, I/R injury reduces miR-21 expression, but miR-21 overexpression decreases renal injury and I/R-AKI by reducing proinflammatory factor synthesis and release (Jia et al., 2020). A recent study showed that NF-κB activation and elevated miR-26a-5p levels suppress renal inflammation by inhibiting IL-6 (Chen et al., 2022). These findings indicate that specific miRNAs act as protective factors and valuable biomarkers for AKI.

#### 3.2.2 miRNAs as damaging factors

Conversely, certain miRNAs overexpression can activate inflammatory pathways and accelerate AKI progression. According to a large dataset study, serum miR-452 levels are elevated in SA-AKI patients, demonstrating 63.83% diagnostic sensitivity. These levels show a positive correlation with serum creatinine and urinary miR-452 (Liu et al., 2020a). Similarly, increased blood miR-210 and miR-494 levels predict poor AKI patient prognosis (Lin et al., 2019).

Animal and cellular studies provide mechanistic insights. In LPS-induced SA-AKI rats, elevated miR-128-3p levels correlate with increased inflammatory mediators and apoptosis. Notably, inhibiting miR-128-3p exacerbates inflammation (Wang L. et al., 2020). miR-376b collaborates with NF-κB to promote cell death in mice, and its decrease in SA-AKI mice’s urine and renal tubular cells is linked to NF-κB activation (Liu et al., 2020b). miR-543 and miR-199b-3p are upregulated in serum, renal tissues, and cells, worsening AKI inflammation and cell death (Zhang et al., 2022; Tian et al., 2021) (Table 2).

**TABLE 2:** The possible molecular mechanisms and functional roles of miRNA in AKI.

miRNA	Sample type	Expression	Functional role	Signaling pathway/target gene	References
miR-22-3p	Kidney tissue and cells	Downregulates	Inhibits inflammation and apoptosis	miR-22-3p/PTEN	Wang et al. (2020a)
miR-93	Kidney tissues and cells	Downregulated	Inhibits apoptosis and inflammatory reactions	PTEN/AKT/mTOR	Zhan et al. (2021)
miR-30c-5p	Renal tissues and cells	Downregulation	Inhibits cellular pyroptosis	miR-30c-5p/TXNIP	Li et al. (2021)
miR-223-3p	Kidney tissues and cells	Downregulated	Inhibition of cellular pyroptosis	NLRP3/Caspase-1/IL-1β	Gao et al. (2022a)
miR-574-5p	Cellular	Downregulates	Increased cell viability	Scr, Cys-C, KIM-1	Liu et al. (2021c)
miR-21	Kidney tissues and cells	Downregulates	Reduces inflammatory response	miR-21/CCR7	Jia et al. (2020)
miR-26a-5p	Kidney tissues and cells	Downregulates	Inhibition of renal inflammatory response	NF-κB/miR-26a-5p/IL-6	Chen et al. (2022)
miR-128-3p	Renal tissues and cells	Upregulates	Promotes inflammatory infiltration, increases inflammatory factor expression and decreases cell viability	miR-128-3p/NPR1	Wang et al. (2020b)
miR-452	Serum, urine and cells	Upregulates	Induction of septic AKI	NF-κB	Liu et al. (2020a)
miR-210, miR-494	serum	Upregulation	Induces septic AKI	--	Lin et al. (2019)
miR-376b	Urine and cells	Upregulates	Promotes inflammation and cell necrosis, exacerbates kidney injury	NF-κB/miR-376b/NFKBIZ	Liu et al. (2020b)
miR-543	Cellular	Upregulated	Promotes inflammation and apoptosis	miR-543/Bcl-2	Zhang et al. (2022)
miR-199b-3p	Kidney tissues and cells	Upregulated	Promotes renal tubular cell edema and necrosis, inflammatory infiltration	miR-199b-3p/Nrf2	Tian et al. (2021)

Despite these findings, significant gaps remain. The origin of circulating miRNAs—whether from kidney leakage or active signaling—requires further investigation. Species differences, such as miR-376b′s divergent trends in mice and humans, highlight the need for cross-species validation. Most mechanistic insights, including miR-376b/NF-κB interactions, derive from cellular studies and require human tissue validation. Future research should focus on validating these miRNAs in larger human cohorts and exploring their therapeutic potential. Addressing these gaps will enhance our understanding of miRNAs in AKI and facilitate their translation into clinical applications.

MiRNAs play a complex dual role in AKI. By targeting specific genes, they regulate inflammation and cell death, exhibiting either protective effects or disease-promoting activities. While current evidence supports the potential of miRNAs in AKI diagnosis and prognosis, further research is required to fully elucidate their mechanisms of action and origin prior to clinical application. Moreover, the consistency of data across species requires additional validation. Future studies should aim to confirm the roles of these miRNAs in larger patient populations and investigate their potential as therapeutic targets, with the goal of achieving new breakthroughs in AKI treatment.

### 3.3 circRNAs

Because circRNAs are more stable than other non-coding RNAs, they are becoming more and more popular as indicators for diagnosis and prognosis, such as miRNAs. Their differential expression in AKI and association with competitive endogenous RNAs make circRNAs promising candidates for therapeutic targeting. To facilitate circRNA-based therapies, new delivery methods are necessary for effective translation into target organs. As our understanding of circRNA’s role in AKI pathophysiology grows, it may significantly impact the assessment and management of the condition.

#### 3.3.1 circRNAs as protective factors

Some circRNAs function as protective factors in AKI, delivering protective signals in blood tests and acting as cellular shields. For example, circ-0091702 levels decrease in the serum of LPS-AKI patients. When active, it serves as a sponge for miR-545-3p, protecting THBS2 and shielding kidney cells from damage (Tan and Bei, 2021). Similarly, circ-YAP1 levels decline in the serum of AKI patients and in injured cells. Enhancing circ-YAP1 helps mitigate fibrosis and inflammation via the miR-21-5p/PI3K pathway (Huang et al., 2020).

These circRNAs also function as cellular guardians. circ-VMA21 exerts protective effects in HK-2 cells by regulating the miR-7-5p/PPARA axis, thereby combating oxidative stress and cell death (Wang et al., 2022). circ-PRKCI binds to miR-106b-5p, freeing GAB1 to aid in cell repair (Xiong et al., 2021). Additionally, circ-0068888 and circ-0008882 jointly inhibit inflammation by respectively targeting miR-21-5p and miR-155-5p (Wei et al., 2021; You and Kuang, 2023).

However, most evidence originates from cellular studies rather than patient-based research. For instance, the protective roles of circ-VMA21 and circ-PRKCI have been predominantly demonstrated in cell models. While the decrease of circ-YAP1 in serum suggests its potential as an AKI biomarker, large-scale patient studies are still required to confirm this.

In summary, these circRNAs show potential as protective factors in AKI, but further validation in patient cohorts is necessary to solidify their role as diagnostic tools.

#### 3.3.2 circRNAs as damaging factors

Some circRNAs act as damaging factors in AKI, serving as both circulating biomarkers and intracellular molecular saboteurs. Clinical studies have shown that circ-0020339 is significantly upregulated in the serum of patients with sepsis-associated AKI (SA-AKI), and its levels correlate with creatinine concentrations. Silencing circ-0020339 improves survival in murine models and reduces LPS-induced cell death and inflammation in HK-2 cells, highlighting its potential as a diagnostic biomarker (Wang et al., 2023). Similarly, the elevation of circ_0114427 in patient blood worsens inflammation through the miR-495-3p/TRAF6/NF-κB axis (Xu L. et al., 2022).

At the cellular level, these circRNAs drive renal injury through distinct pathological mechanisms. Circ-RASGEF1B promotes inflammation and apoptosis via the miR-146a-5p/Pdk1 axis, directly reducing tubular cell viability (Cao et al., 2021). Circ-UBE2D2 sponges miR-370-3p to upregulate NR4A3, triggering apoptosis in LPS-exposed HK-2 cells (Huang and Zheng, 2022). Circ-AKT3 impairs cellular repair by disrupting the miR-144-5p/Wnt/β-catenin pathway, exacerbating ischemic tubular damage (Xu Y. et al., 2022). Circ-ITGB1, activated by GATA-binding protein, induces inflammatory responses through the miR-328-3p/PIM1 axis (Gao Y. et al., 2022). The mouse-specific mmu_circ_0000943 aggravates oxidative stress and apoptosis by sponging miR-377-3p and overexpressing Egr2 (Huang et al., 2022). Circ-SNRK activates MAPK signaling (p-JNK/p38), promoting inflammation and apoptosis in renal tissues (Meng et al., 2022). Notably, context-dependent roles exist. For example, circ_0023404 suppresses inflammation by targeting IL-6R in some settings (Xu et al., 2021), yet may promote damage in others—underscoring microenvironmental influences on circRNA functionality (Table 3).

**TABLE 3:** The possible molecular mechanisms and functional roles of circRNA in AKI.

circRNA	Sample type	Expression	Functional role	Signaling pathway/target gene	References
circ-0091702	Cellular	Downregulates	Attenuates cellular damage	miR-545-3p/THBS2	Tan and Bei (2021)
circ-VMA21	Cells	Downregulates	Inhibits oxidative damage, inflammatory processes, and cell death	miR-7-5p/PPARA	Wang et al. (2022)
circ-PRKCI	Cells	Downregulates	Inhibits apoptosis, inflammation and oxidative stress	miR-106b-5p/GAB1	Xiong et al. (2021)
circ-YAP1	Serum and cells	Downregulates	Reduces inflammatory response, inhibits cellular damage	miR-21-5p/PI3K/Akt/mTOR	Huang et al. (2020)
circ-0068888	Cellular	Downregulation	Inhibits inflammatory response and oxidative stress	miR-21-5p/NF-κB	Wei et al. (2021)
circ-0008882	Cells	Downregulates	Attenuates cellular damage	miR-155-5p/PDE7A	You and Kuang (2023)
circ-RASGEF1B	cellular	Upregulates	Encourages inflammatory cell response and cell death	MicroRNA-146a-5p/Pdk1	Cao et al. (2021)
circ-UBE2D2	Cells	Upregulates	Inhibits cell viability, promotes apoptosis	miR-370-3p/NR4A3	Huang and Zheng (2022)
circ-0020339	Serum and cells	Upregulates	Promotes apoptosis and inflammatory responses	miR-17-5p/IPMK TRAF6/p-AKT/p-IKK/p-IκBα/p-p65	Wang et al. (2023)
circ_0023404	Cell	Upregulation	Stimulates secretion of inflammatory factors and promotes inflammatory responses	miR-136/IL-6R	Xu et al. (2021)
Circ_0114427	Serum and cellular	Upregulates	Inhibits cell viability and promotes inflammation responses and necrosis	miR-495-3p/TRAF6/NF-κB/p65	Xu et al. (2022a)
Circ-AKT3	Renal tissues and cells	Upregulated	Promotes oxidative damage and apoptosis	miR-144-5p/Wnt/β-catenin	Xu et al. (2022b)
circ-ITGB1	Cells	Upregulated	Promotes inflammation responses and necrosis	miR-328-3p/PIM1	Gao et al. (2022b)
mmu_circ_0000943	Kidney tissue and cells	Upregulated	Stimulates inflammatory response and promotes apoptosis	miR-377-3p/Egr2	Huang et al. (2022)
circ-SNRK	Kidney tissue, serum and cells	Upregulates	Promotes inflammatory factor secretion and apoptosis	MAPK/p-JNK, p-38	Meng et al. (2022)

In summary, these circRNAs play a significant role in AKI by influencing various pathological processes such as inflammation, apoptosis, and cellular repair. Their dual role as biomarkers and active participants in disease progression makes them potential targets for diagnostic and therapeutic strategies in AKI. However, further research is needed to fully elucidate their mechanisms and clinical applications.

### 3.4 Other types of ncRNA in AKI

In addition to lncRNAs, other non-coding RNAs, such as small nucleolar RNA (snoRNA) and small nuclear RNA (snRNA), warrant exploration for their potential roles in acute kidney injury (AKI) (Zhu et al., 2024; Liu et al., 2019). SnoRNAs primarily function in the chemical modification of ribosomal RNA (rRNA) and have been implicated in various cellular processes, including gene expression regulation. Emerging evidence suggests that specific snoRNAs may influence renal cell survival and apoptosis during AKI by modulating rRNA stability and facilitating ribosome biogenesis. Changes in snoRNA expression profiles have been linked to inflammatory responses in kidney tissues, indicating a potential role in AKI pathophysiology.

In a similar vein, snRNAs are necessary for pre-mRNA splicing, which results in the production of functional mRNA. Anomalies in the splicing of genes related to stress responses and renal function may arise from dysregulation of snRNA, contributing to AKI development. However, research on snoRNAs and snRNAs in AKI is limited, and this section does not serve as the primary focus of this study. Future investigations will aim to provide further insights into the contributions of these ncRNAs, enhancing our understanding of renal injury mechanisms and improving clinical management strategies.

## 4 Developments in the research of natural medicines controlling ncRNA for the therapy of AKI

**FIGURE 2:**
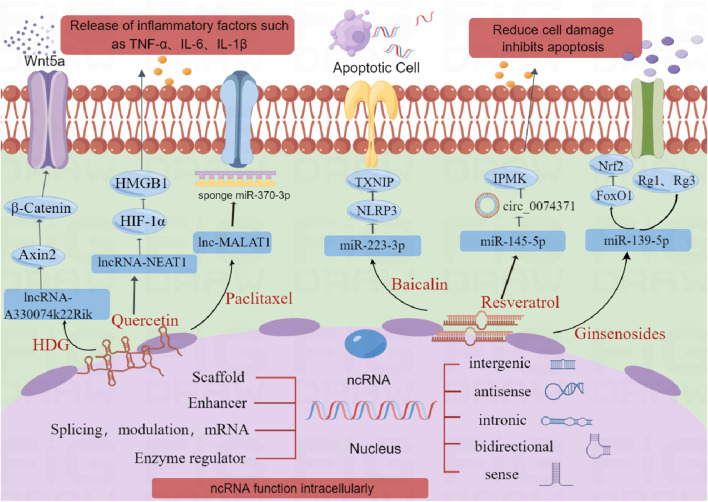
Illustration of Non-coding RNA Functions and Natural Medicine Regulations in Acute Kidney Injury. The figure illustrates the roles of natural medicines in modulating ncRNAs for the therapy of acute kidney injury (AKI). Various natural compounds, including quercetin, paclitaxel, ginsenosides, baicalin, and resveratrol, are shown to influence ncRNA activity. These compounds can affect lncRNAs and circRNAs, which in turn regulate inflammation, apoptosis, and other cellular processes. (Created with FigDraw).

Research on non-coding RNAs (ncRNAs) is gaining traction in the field of traditional Chinese medicine (TCM). Various natural compounds and herbal extracts show promise in mitigating renal injury and apoptosis by modulating ncRNA activity (Figure 2). This section summarizes the effects of these herbal extracts on protein expression via ncRNA and their potential for treating acute kidney injury (AKI).

### 4.1 Quercetin

Quercetin, abundant in Chinese herbs like Radix Bupleuri and mulberry leaf, offers anti-inflammatory, anti-cancer, and antioxidant benefits. It protects kidneys by raising glutathione (GSH) levels and cutting lipid ROS and malondialdehyde, thus easing ischemia/reperfusion (I/R) -induced AKI. Quercetin also inhibits HIF-1α in the NEAT1/HMGB1 pathway, reducing cell damage. This suggests quercetin could be a new therapeutic target (Wang Y. et al., 2020; Luo et al., 2022).

It is interesting to note that quercetin’s ability to modulate the NEAT1/HMGB1 pathway may have broader implications for AKI treatment. By targeting this pathway, quercetin not only reduces oxidative stress but also attenuates inflammation, which are both critical factors in the progression of AKI. However, further research is required to fully understand the extent of quercetin’s effects and its potential for clinical application.

### 4.2 Paclitaxel

Paclitaxel, the first plant-derived chemotherapeutic agent from the redbud tree bark, is widely used in oncology. Recent studies show it enhances cell proliferation and reduces pro-inflammatory cytokines like TNF-α, IL-1β, and IL-6. Paclitaxel mitigates LPS-induced AKI by altering the lnc-MALAT1/miR-370-3p/HMGB1 axis, suggesting a potential therapy for sepsis -associated AKI (Xu et al., 2020).

Paclitaxel’s role in regulating the lnc-MALAT1/miR-370-3p/HMGB1 axis highlights its potential as a therapeutic agent for AKI. The ability to modulate this specific axis could provide a targeted approach to reducing inflammation and preventing further kidney damage in patients with sepsis. However, the long-term effects and safety of paclitaxel in AKI treatment need to be further evaluated.

### 4.3 Ivy saponin elements

Ivy saponinogenin (HDG), a pentacyclic triterpenoid saponin in astragalus mongholicus, shows strong anti-inflammatory properties across various diseases. Kehuan Xie used HDG to treat LPS-induced kidney inflammation in renal tubular epithelial cells, both *in vivo* and *in vitro*. Transcriptome sequencing revealed significant changes in lncRNA expression, especially lncRNA-A330074k22Rik (A33). Ivy saponins also reduce cisplatin-induced renal injury via the A330074k22Rik/Axin2/β-catenin pathway (Xie et al., 2022).

The significant changes in lncRNA expression observed with HDG treatment indicate a potential therapeutic mechanism for ivy saponins in AKI. By targeting specific lncRNAs and their associated signaling pathways, ivy saponins may offer a novel approach to reducing renal injury.

### 4.4 Baicalin

Baicalin, a bioactive flavonoid from *Scutellaria baicalensis*, is known for its anti-inflammatory, diuretic, and antibacterial properties. It significantly improved HK-2 cell viability and reduced levels of inflammatory markers such as TNF-α, IL-1β, and IL-6. Baicalin downregulates cyclooxygenase-2, inducible nitric oxide synthase, TXNIP, NLRP3, and miR-223-3p. Its mechanism likely involves suppressing the TXNIP/NLRP3/miR-223-3p pathway (Sun et al., 2020).

Baicalin’s multi-faceted approach to reducing inflammation and improving cell viability makes it a promising candidate for AKI treatment. By targeting multiple components of the inflammatory pathway, baicalin may provide a more comprehensive therapeutic effect. However, the clinical feasibility of baicalin as a treatment option in AKI patients remains to be determined through further studies.

### 4.5 Resveratrol

Resveratrol, present in over 70 plant species, has diverse biological effects. It lowers TNF-α in AKI rat models and reduces pro-inflammatory cytokines. Resveratrol inhibits MALAT1 expression, protecting kidneys via the lncRNA MALAT1/miR-205 axis (Wang B. et al., 2021). It also regulates pathways linked to circ_0074371, which is elevated in LPS-treated HK-2 cells and AKI patients. Resveratrol exerts nephroprotective effects through the circ_0074371/miR-145-5p/IPMK pathway, reducing cellular apoptosis and oxidative stress (Zhu and Wu, 2024).

Resveratrol’s ability to modulate both lncRNA and circRNA pathways offers a dual therapeutic approach for AKI. By targeting these specific RNA networks, resveratrol may provide a more effective treatment strategy. However, the translation of these findings into clinical practice requires additional research to confirm resveratrol’s efficacy and safety in human subjects.

### 4.6 Ginsenoside

Ginsenosides, the main active components of ginseng, are used in TCM for their anti-cancer and immunomodulatory effects. Ginsenoside Rd protects against ischemia/reperfusion injury by suppressing FoxO1 and stimulating the Nrf2 antioxidant pathway. Other ginsenosides like Rg1 and Rg3 decrease renal injury markers such as creatinine and urea nitrogen (Yao et al., 2022; Chang et al., 2023).

Ginsenosides’ dual action of reducing renal injury markers and protecting renal structures makes them potential therapeutic agents for AKI. Their ability to modulate both the FoxO1 and Nrf2 pathways suggests a comprehensive approach to reducing kidney damage. However, the specific mechanisms and optimal dosages for different ginsenosides in AKI treatment need to be further explored.

In summary, several ncRNAs show potential as biomarkers and therapeutic targets in AKI. Natural compounds like quercetin, paclitaxel, and resveratrol modulate specific RNA networks, offering new therapeutic approaches for AKI. However, the connection between natural medicine and ncRNA regulation needs more concrete evidence. Future studies should build on these examples and explore additional natural compounds that can specifically target ncRNA networks involved in AKI.

## 5 Discussion

Non-coding RNAs (ncRNAs) have shown significant promise as biomarkers and therapeutic targets for acute kidney injury (AKI). However, there are also limitations and inconsistencies in the current research that need to be critically evaluated.

### 5.1 Challenges in clinical applicability of ncRNA biomarkers

The translation of ncRNA biomarkers from preclinical models to clinical practice faces several unresolved challenges. In terms of sensitivity and specificity, such as miR-452 in serum showing 63.83% sensitivity for AKI diagnosis (Liu et al., 2020a), this finding lack validation in large human cohorts. Moreover, cross-reactivity with non-renal conditions like systemic inflammation may compromise the specificity of ncRNA biomarkers.

Diagnostic standardization is another major challenge. Current AKI diagnostics mainly rely on serum creatinine and urine output. However, ncRNA-based detection requires standardized protocols for sample collection, RNA isolation, and quantification. The variability in techniques, such as RNA-seq versus qPCR, contributes to inconsistent thresholds, as seen in the upregulation of circ-0020339 in sepsis-associated AKI (SA-AKI) (Wang et al., 2023).

Additionally, the dynamic range of ncRNAs poses a challenge. The levels of ncRNA may fluctuate with different stages of AKI; however, longitudinal studies defining its temporal dynamics are still scarce.

### 5.2 Mechanistic gaps and conflicting findings

There are several pathways where the mechanistic depth is lacking or contradictory roles are observed. Crucially, the dysregulation of ncRNAs is intrinsically linked to the development of hallmark AKI pathological features, including acute tubular injury (ATI), interstitial inflammation, and the initiation of maladaptive repair processes that can lead to fibrosis. The dualistic functions of ncRNAs are evident. For instance, GAS5 acts as a protective factor in SA-AKI by inhibiting pyroptosis via the miR-579-3p/SIRT1 pathway, yet it promotes apoptosis in ischemia/reperfusion (I/R)-AKI through the miR-21/TSP-1 pathway (Ling et al., 2021; Geng et al., 2020). This discrepancy may arise from model-specific stressors, such as sepsis versus ischemia, highlighting the need for context-dependent pathway validation.

Similarly, miR-128-3p exacerbates inflammation in lipopolysaccharide (LPS)-induced AKI but attenuates injury in I/R models via JAK/STAT modulation (Xie et al., 2020; Wang L. et al., 2020). These contrasting roles suggest that the microenvironment significantly influences ncRNA functionality and its downstream pathological consequences. The net effect on tubular cell survival, inflammatory infiltration, and interstitial damage varies depending on the ncRNA and the injury context.

Furthermore, the link between ncRNA dysregulation and the progression towards fibrosis, although more characteristic of CKD, is an emerging concern in AKI recovery. Some ncRNAs implicated in sustained inflammation and impaired tubular repair could potentially lay the groundwork for interstitial fibrosis if the injury persists or repair is dysregulated. While not the primary focus of acute injury, understanding how early ncRNA changes might predispose to or initiate fibrotic pathways is a critical mechanistic gap.

Unresolved issues also exist in pathway crosstalk relevant to pathology. NLRP3 inflammasome activation is frequently linked to lncRNAs and miRNAs (Zhang X. et al., 2021; Gao M. et al., 2022). However, upstream regulators and feedback loops remain underexplored. The Wnt/β-catenin pathway is also variably reported as protective or damaging, indicating cell-type-specific signaling outcomes that influence tubular repair versus dysrepair and potential fibrotic transformation (Li et al., 2020; Liu D. et al., 2021).

In summary, while numerous ncRNAs are dysregulated in AKI, explicitly linking their altered expression to the causation and progression of specific pathological hallmarks—such as acute tubular necrosis/apoptosis, interstitial inflammatory cell influx, oxidative stress-mediated damage, and the nascent stages of fibrosis—remains an area requiring deeper investigation.

### 5.3 Barriers to therapeutic translation

When it comes to therapeutic translation, there are several barriers to consider. Delivery and specificity are key challenges. While natural medicines like baicalin and resveratrol have shown efficacy in modulating ncRNAs *in vitro*, targeted delivery to renal tubules *in vivo* remains unresolved (Sun et al., 2020; Wang B. et al., 2021; Zhu and Wu, 2024). Additionally, off-target effects of ncRNA inhibitors, such as antagomiRs, may disrupt physiological gene networks.

Another barrier is compensatory mechanisms. Silencing a single ncRNA often does not fully rescue AKI phenotypes, suggesting redundant roles within competing endogenous RNA (ceRNA) networks. This implies that combinatorial targeting strategies may need to be investigated.

### 5.4 Future perspectives

Despite the limitations and challenges, the potential of ncRNAs in AKI research remains promising. In the future, we need large-scale trials to validate ncRNA therapies and develop better delivery systems. We must deeply explore ncRNA regulatory networks to understand AKI better and find new treatments. Also, it's key to link ncRNA signatures to specific AKI pathological features. Using techniques like *in situ* hybridization on kidney tissue can map ncRNA expression in damaged areas, inflammation, or early fibrosis. This will clarify ncRNAs’ role in AKI pathology and improve their use as biomarkers.

## 6 Summary

This review has provided a comprehensive overview of the role of ncRNAs in AKI, focusing on lncRNAs, circRNAs, and miRNAs. We have highlighted their dualistic nature in AKI pathogenesis, acting as both protective and detrimental factors. The integration of natural medicine with ncRNA regulation offers a novel perspective for the development of therapeutic strategies. Moreover, it is important to critically evaluate the limitations and inconsistencies in the current research. Future studies should aim to bridge the translational gap between preclinical models and clinical practice, address the challenges in using ncRNAs as biomarkers, and explore the potential of ncRNA-targeting natural compounds through well-designed clinical trials. Furthermore, the application of bioinformatics and systems biology approaches will be instrumental in unraveling the complex ncRNA networks in AKI. Continued research in this field has the potential to significantly advance our understanding of AKI pathogenesis and pave the way for innovative diagnostic and therapeutic strategies. Ultimately, this could enhance patient outcomes and provide a clearer understanding of the complex mechanisms governing kidney injury.

Research into herbal treatments in the context of ncRNA regulation in AKI is still in its early phases. Current research primarily focuses on isolated compounds and extracts derived from Chinese herbs, with less emphasis on complex herbal formulations or proprietary Chinese medicines that are frequently employed in clinical settings. This gap highlights the need for more comprehensive studies that investigate the synergistic effects of multi-ingredient herbal formulations on ncRNA regulation and their subsequent impact on AKI outcomes. Furthermore, bioinformatics tools can play a crucial role in advancing this field. By predicting gene interactions with ncRNAs, bioinformatics approaches could accelerate the identification of potential biomarkers and therapeutic targets. These insights can lead to the development of innovative diagnostic applications and targeted treatments for AKI.
